# Assessment of speech sound disorders: Clinical experiences of Speech-language pathologists in Iran

**DOI:** 10.1371/journal.pone.0310885

**Published:** 2024-12-16

**Authors:** Mersede Imani-Shakibayi, Talieh Zarifian, Mina Fotuhi, Michelle Pascoe, Mojdeh Khorsand-Moghadam, Fatemeh-Zahra Bazdar

**Affiliations:** 1 Department of Speech Therapy, Faculty of Rehabilitation Sciences, University of Social Welfare and Rehabilitation Sciences, Tehran, Iran; 2 Pediatric Neurorehabilitation Research Center, University of Social Welfare and Rehabilitation Sciences, Tehran, Iran; 3 Department of Speech Therapy, Faculty of Rehabilitation Sciences, Iran University of Medical Sciences, Tehran, Iran; 4 Department of Health and Rehabilitation Sciences, University of Cape Town, Cape Town, South Africa; Education University of Hong Kong, HONG KONG

## Abstract

**Background:**

Speech-language pathologists (SLPs) in non-English speaking countries face challenges when assessing children for speech sound disorders (SSD). Exploring their clinical challenges in service delivery–along with their problem-solving approaches–may contribute to the development of instruments for clinical use in such settings.

**Aim:**

The study aimed to explore assessment methods used by Iranian SLPs to identify children with SSD.

**Methods:**

A cross-sectional survey took place with 113 SLPs working with children with SSD. The survey comprised 36 items grouped into four main themes–service delivery, types of assessments procedures, challenges of evaluating SSD, and ways of keeping up to date. Results were analysed based on participants’ levels of education and experience.

**Results:**

Participants used Persian as the primary language in their work, and over 70% provided services in a private setting. Most participants reported having between 10–30% of clients with SSD in their caseload. Most (73.5%) SLPs involve parents in completing the history form. They favour informal traditional articulation tests, with approximately half the sample selecting this approach from the 14 options presented to them, especially those with bachelor’s degrees and minimal experience. More experienced SLPs (62.7%) chose the Polysyllabic Words Test. Assessment, whether formal or informal, was found to be time-consuming by the majority of respondents (18.6% always, 42.5% often). Self-study (65.5%) and taking part in workshops/webinars/journal clubs (60.1%) were ways to keep their knowledge current.

**Conclusion:**

Our findings are similar to other international survey results on SSD evaluation. Although many Iranian SLPs use informal traditional articulation tests, such assessments are limited in their analysis and the diagnostic information provided. The findings emphasise the need for clear phonological assessment guidelines, valid and reliable ‘clinician friendly’ instruments, equitable distribution of tools across the country, and suitable tools for bi/multilingual communities.

## Introduction

Children with speech sound disorder (SSD) comprise a substantial proportion of caseloads in clinical settings [1], and Shriberg and Kwiatkowski (1994) stated that 7.5% of children between 3 and 11 years of age are affected [2]. The reported prevalence of SSD varies between 1.3% to 22.9% [3–6], and in Iran specifically, reports range from 6.5% to 13.8% in primary school students [7, 8]. Attempts to categorise children with SSD have led to identification of three subgroups: phonological, articulation-based and motor planning/programming subgroups [9–11]. However, further research is needed regarding consensus-based identification systems and an agreed SSD classification system to help Speech-language pathologists (SLPs) select suitable assessment tools and appropriate treatment approaches [12].

### Service delivery

Many factors influence SLPs’ choice of assessments when evaluating children’s speech, including caseload size, work setting, level of SLP education, adherence to guidelines, and client language [12–15]. Surveys have addressed service delivery options, such as assessment location (e.g., hospital, clinic, school), service model (e.g., individual/group therapy, home visits), people involved in the process (e.g., clinician, parents, caregiver), and language/s used by clients and clinicians. However, reports on SLP’s services to children with SSD and their families are limited and conflicting. The most common method of service delivery in Australia is individual therapy in clinics, while in the US, SLPs provide services in preschools and schools as a first line of intervention [12, 13, 15], and also work in hospitals, private clinics, and schools. Swedish SLPs mainly see children with SSD in hospitals [14], while South African [16] and Australian SLPs [12] often encounter children with SSD in private practice.

### Assessing speech sound production

Assessment is a critical and time-consuming activity, reported by SLPs to take approximately 2 to 2.5 hours [13]. Assessment involves three phases. *Pre-assessment* activities typically include conducting interviews with parents and auditory screening [12, 13]. During *direct assessment* evaluation processes are used to profile children’s speech production either with or without parent involvement [16, 17]. Single-word tests are widely used for sampling, while eliciting spontaneous speech as an ecological speech sample is recommended [12, 16, 18, 19]. Standardised single-word tests are the most common tools used by clinical practitioners [12–14, 17, 20, 21]. Many have been designed for monolingual English-speakers, and SLPs working with non-English speaking children must adapt them or develop assessments for the languages routinely spoken in their region [12, 13, 21, 22]. English-speaking clinicians often compensate for this shortage by using formal English tests to evaluate children’s speech production, regardless of the child’s first language and without taking cultural perspectives into account [13, 14, 21, 23]. In *post-assessment* activities, relational and independent phonological analyses are used to complement speech sampling along with documenting clinical findings of all assessment phases.

A literature review of the types of assessment frequently used by SLPs showed discrepancies between English- and non-English speaking countries. Most British SLPs use phonology screening tests to determine sounds that children struggle with [24]. US SLPs (74%) use standardised single-word speech tests, stimulability testing, hearing screening and oral-motor examination to estimate intelligibility [13]. The priority of Australian SLPs is connected speech sampling followed by informal single-word tests to determine sound errors [12], and standardised tests are used by a smaller proportion (11%) who specifically mentioned formal tests with Australian normative data. Farquharson and Tambyraja (2019) emphasised the need for US school-based SLPs to conduct standardised testing, elicit speech samples, and undertake oral mechanism examinations when evaluating children for SSD [15].

Dutch and Swedish SLPs were surveyed to provide information on non-English speaking countries. The Nederlands Articulatie Onderzoek [25] and Hodson Assessment of Phonological Patterns [26] were the most popular assessments used by Dutch SLPs, but connected speech samples were not widely mentioned [20]. Wikse Barrow et al. (2021) revealed Swedish SLPs’ tendency to use standardised tests for evaluation of speech output, phonological awareness, speech consistency and oral-motor functioning [14]. The only research regarding Iranian SLPs’ assessment of children with SSD was an investigation of practitioner viewpoints on childhood apraxia of speech (CAS) [27]. The results of that study were consistent with other studies indicating a growing inclination to use more informal tests in the absence of applicable specifications to evaluate CAS [18]. Wong et al. (2023) recently reported that in the absence of standardised measures for differential diagnosis of CAS by SLPs in Hong Kong, speech sampling, oral motor examination and motor skills tasks were implemented instead [28].

### Challenges of evaluating SSD

Practitioners face challenges in assessing children with SSD. Caseload size, limits on child cooperation and paperwork overload are frequently cited difficulties [12, 13, 18]. A commonly reported issue in non-English speaking countries is the lack of linguistically appropriate assessment tools for clients, and inadequate support or training to meet the needs of multilingual populations in both assessment and therapeutic procedures [13, 22]. Swedish clinicians report difficulty in the differential diagnosis of SSD subtypes as the most significant challenge of assessment [14].

### Keeping up to date with the evidence base

SLPs must remain up to date to practice effectively in the field. Undergraduate courses are a common and accessible way to obtain basic information in the SSD domain. However, the process of science development is dynamic, and new research evidence is being produced all the time. Thus, SLPs who graduated several years ago need to keep incorporatingnew evidence in a continuous manner. To gain a deeper understanding of clinical implications, SLPs may read journal articles and textbooks, attend workshops, and participate in continuing professional development programs. Inquiring about ways to stay up-to-date and addressing related difficulties is helpful for planning these activities. In US and Australian studies, undergraduate/graduate courses and workshops influenced SLPs’ clinical practice [13], and self-study (reading journals/book chapters) was popular. Despite being native English speakers, Australian SLPs expressed difficulty in understanding the complexity of the literature and accessing relevant journals [12].

### Evaluating SSD and available assessment tools in Iran

Single-word speech samples, conversational speech sampling, and informal tests are widely used assessment methods. Clinicians in previous studies showed variability in the use of assessments and service delivery, which–on the one hand–reveals their flexibility in selecting appropriate assessment tools, but on the other hand, may indicate a lack of knowledge or confidence in choosing appropriate assessments.

In Iran, the Ministry of Health and Medical Education oversees 11 Medical Sciences Universities that train speech therapy students at different academic levels (BS, MS, and PhD). These universities admit approximately 200 bachelors’, 30–35 masters’, and 10 doctoral students annually through a nationwide exam. On graduation, bachelor’s students are required to complete a two-year internship at government centres to obtain clinical competency approval from the Ministry of Health. Registration with the Medical Council and authorization to work in private centres is mandatory.

Healthcare facilities that offer speech therapy services include government centres, such as clinics, hospitals, and special education schools, and private centres, such as offices and charitable institutions serving individuals with special needs. Statistics from the Medical Council of Iran indicate that of 3931 members of the SLP organization, 878 hold a professional qualification license. The Iranian Speech Therapy Association and the Ministry of Health review SLPs’ activities and address patient complaints. Although there are no specialised healthcare centres for children with SSD in Iran, both government and private healthcare facilities with speech therapy clinics offer services to children with SSD.

Persian is the official language spoken in Iran, but development of valid and reliable speech assessment tools in this language remains limited. Attempts to design, develop and introduce speech assessment tools in Persian [29–35] and other commonly spoken languages of the country (e.g., Azeri-Azerbaijani Turkish, Kurdish and Laki) have increased during the last decade [36–38]. An informal Traditional Articulation Test (TAT) based on Templin and Darley (1960) [39] was developed and adapted for Persian, and has been widely used since the emergence of Speech Therapy in the country. Of the 17 assessment tools reported in present study, the only published test with normative data is the Persian Diagnostic Evaluation of Articulation and Phonology (P-DEAP), which includes diagnostic screening, phonological, articulation, inconsistency and oral-motor subtests [40–42]. This tool has been introduced to Iranian SLPs in SSD undergraduate courses, workshops and webinars. However, whether it is widely used in clinical contexts remains unclear.

Information regarding the evaluation methods used by Iranian SLPs to diagnose SSD is lacking. Comparing Iranian SLP’s practices with clinical advances in the field and other research reports will elucidate clinicians’ fundamental achievements, paving the way for designing relevant tools, contributing to overcoming challenges in the evaluation process, and introducing Persian tools in the SSD field for use in diverse contexts. The present study aimed to provide more detail on SLP practices for speech sound assessment in Iran. Our research questions were as follows: 1) What are the frequency and types of assessment methods used by Iranian SLPs? It was hypothesized that the P-DEAP assessment is the most used tool for assessing SSD. 2) How do Iranian SLPs provide SSD evaluation services? 3) What challenges do Iranian SLPs face during the evaluation of SSD? 4) How do participants ensure their knowledge and skills remain current? and 5) Is there a correlation between the assessments chosen by SLPs and their level of education, experience, and confidence?

## Materials and methods

The study received ethical approval from the University of Social Welfare and Rehabilitation Sciences Research Ethics Committee (approval number: IR.USWR.REC.1401.141). A covering letter and informed consent form containing information about the study were provided as the first page of the online and printed questionnaire. The questionnaires did not include any identifying data that directly or indirectly could be attributed to participants. The respondents who filled out printed questionnaires signed the consent form, while those who completed the online questionnaire were required to submit their answers to be included in the analysis. Therefore, all participants gave their written informed consent to participate in the study and were informed that their responses would remain anonymous.

### Participants

The target population of this study was Iranian SLPs with medical council membership and at least one year of clinical experience in one of the speech therapy centres assessing at least one case of SSD. A total of 113 participants filled out the questionnaire. Most participants were female (n = 91, 80.5%). The respondents had experience ranging from 1 to 38 years of practice (M = 8.38, SD = 8.67) and working hours from 1 to 70 hours weekly (M = 22.74, SD = 14.36). SLPs with Bachelor degrees (n = 66, 58.4%) were most typical. To identify clinicians’ level of experience, we considered the number of years of practice including hours of weekly activity as an SLP, divided into three levels (less-experienced, mid-experienced and experienced). Demographic details are included in Table 1.

**Table 1:** Demographic information of participants (*N* = 113).

Participant characteristics		Number	%
***Gender***	Female	91	80.5
	Male	22	19.5
***Level of education***	Bachelor of Science (B.Sc)	66	58.4
	Master of Science (M.Sc)	36	31.9
	Doctor of Philosophy (PhD)	11	9.7
***Years of practice***	1–3 years	49	43.4
	4–6 years	19	16.8
	7–15 years	22	19.5
	>16 years	23	20.4
***Hours of practice per week***	1–5 hours	8	7.1
	6–15 hours	30	26.5
	16–40 hours	62	54.9
	>41 hours	11	9.7
***Level of experience***	Less-experienced	23	20.3
	Mid-experienced	31	27.4
	Experienced	59	52.2
***Total***		113	100

### Survey design

The questionnaire was developed through interviews with expert SLPs in SSD by the first author and a review of key literature [13]. The initial version of the questionnaire was prepared and discussed by the first four authors. Then, the face and content validity of the 34 developed questions were evaluated in an expert panel including eight first language Persian- speaking SLPs with academic and clinical experience in SSD. The steering group of SLPs discussed each question in terms of relevance, simplicity and clarity. Eight questions were revised and modified; four questions were added, and two questions were deleted from the survey. The prepared draft of the questionnaire was reviewed by the fourth author, an expert SLP in the field of SSD. Subsequently, minor changes were made to the formatting and wording of the questions. Ten Persian-speaking SLPs with bachelor’s degrees were recruited for the pilot study using the final version of the questionnaire, and provided feedback about its readability and general format.

The final draft of the questionnaire included 36 questions covering four separate sections: A) Demographic information, B) Service delivery, C) Assessment, and D) Intervention/Treatment. Participants were provided with a summary of the research and instructions on how to complete the survey.

The demographic section included multiple-choice questions regarding gender, education, years of experience, hours a week engaging in clinical practice, main field of expertise and age groups of the children seen (Q1–Q6). The second part (service delivery) included seven questions divided into four subsections of city and language of service delivery, caseload, and practice site (Q7–Q13). The assessment section considered SLPs’ choice of general pre-assessment items (Q14–Q15), direct assessment (Q16) and post-assessment procedure (Q17–Q20). In the pre-assessment section, participants were requested to list three activities they implement before evaluating children’s speech by choosing one answer (“always”, “often”, “sometimes”, “rarely” and “never”). The direct assessment subsection included 16 tests plus one informal widely-used articulation assessment to identify the assessments most prioritised by clinicians. For each item, participants were asked to select if they “always”, “often,” “sometimes,” “rarely,” or “never” used an assessment. In the post-assessment part, participants were asked to choose six speech components used for supplementary analysis (Q17). Three questions regarding clinical records were also included in this part (Q18–Q20). Clinical challenges faced by SLPs during assessment of children with SSD were listed as nine predetermined choices, and participants were asked to choose the frequency with which they experienced them as challenging (Q21). The participants were also asked to rate their level of self-confidence during assessment (Q22). They were asked to indicate the activities that help them remain up to date (Q23), with self-study, participation in workshops and attending graduate/postgraduate courses as possible answers. The final part of the questionnaire included 11 questions regarding treatment (Q24–Q36). S1 Appendix presents the questionnaire.

Responses to the questions regarding the assessment of children with SSD are reported in the present article.

### Procedure

The data were gathered through convenience and snowball sampling methods. The questionnaire was designed in both online and printed form. An online survey was administered via a survey host (https://survey.porsline.ir/) and was available for three months. The survey link was distributed via email and social media such as WhatsApp, Telegram and Instagram. Printed copies were also prepared for distribution to clinicians at workshops presented by the first and second authors on SSD and in their work settings. The questionnaire took approximately 20 minutes to complete.

### Data analysis

The online recorded responses were stored in an Excel file. All the quantitative results were entered in SPSS version 26.0 and were analysed using descriptive statistics to display frequency counts, means and medians. Categorical data comparisons were conducted using the Chi-square test to explore the relationship between type of procedures (pre/direct/post-assessment) and SLPs’ level of experience/education; and between level of confidence and assessment procedures. Spearman’s rank correlation coefficient was used to identify relationships between years of clinical practice or hours of practice per week and types of assessment. The authors asked an open-ended question about the most significant challenges that SLPs encountered when documenting their clinical work. The question was analysed qualitatively, and all responses were coded by the first and third authors. SLPs’ comments were labelled with short phrases and the most frequent main themes were reported in the post-assessment procedure subsection. All statistical analysis was considered significant at the level of α = 0.05.

## Results

Responses of 85 SLPs working in the SSD field were received online and 28 questionnaires were filled out physically. Results are presented in four sections including service delivery, assessing speech sound production, challenges of evaluation and ways of keeping updated.

### Service delivery

The language employed in the work setting, the place of service delivery and caseload are reported in this section.

#### Language

Persian was used by all participants (*N* = 113, 100%) in their work. English (*n* = 11, 9.7%), Azeri-Azerbaijani Turkish (*n* = 10, 8.8%) and Kurdish (*n* = 6, 5.3%) were the most spoken languages after Persian. The other languages/dialects mentioned by Iranian SLPs included Gilaki, Arabic, Baloochi, Lari, Dashti, Turkmen and Qazaq tili (Iranian dialects) and Swedish, Spanish and Turkish.

#### Practice sites

Nine work settings were presented in multiple choice question format. The sites reported were private practice setting (*n* = 81, 71.7%), private hospital (*n* = 3, 2.7%), government school (*n* = 3, 2.7%), private school (*n* = 0, 0.0%), government clinic or hospital (*n* = 39, 34.5%), university hospital (*n* = 31, 27.4%), rehabilitation centre (*n* = 14, 12.4%) and Non-Governmental Organization (NGO) (*n* = 8, 7.1%). The other settings mentioned by SLPs included home visits (*n* = 3, 2.6%) and online service delivery (*n* = 1, 0.9%).

#### Caseload

The percentage of SSD clients reported by SLPs from their clinical records was between 10–30%.

### Assessing speech sound production

The results in this section are grouped into the three phases of assessment (pre-assessment, direct assessment, and post-assessment data analysis/supplementary tasks). The frequency of the assessment tools’ use was determined with a Likert-type scale (from “always” to “never”). The SLPs were invited to answer “How confident do you feel about choosing the assessments most appropriate for diagnosing your clients’ SSDs?” Nearly 60% of the participants indicated that they felt very confident choosing assessments.

#### Pre-assessment procedure

Participants were asked to indicate their use of each of three pre-assessment activities, including conducting interviews with parents, auditory screening and reviewing the case history (Table 2).

**Table 2:** The frequency of pre-assessment activities used by SLPs (*N* = 113).

Pre-assessment activities	Always	Often	Sometimes	Rarely	Never
Interview with parents	99 (87.6%)	11 (9.7%)	3 (2.7%)	0	0
Completing child history form	109 (96.5%)	3 (2.7%)	1 (0.9%)	0	0
Auditory screening	53 (46.9%)	33 (29.2%)	21 (18.6%)	4 (3.5%)	2 (1.8%)

*Parental involvement in assessment procedure*. The respondents were invited to describe the extent of parental involvement in the assessment process with multiple responses accepted. Completing the history form (73.5%) and face to face interviewing (75.2%) were the most frequent activities of parents/caregivers. Only 1% reported “no parental participation” in the assessment procedure (Table 3).

**Table 3:** Parental involvement in assessment of children with SSD as reported by respondents (*N* = 113).

Parental involvement in assessment process	Always	Often	Sometimes	Rarely	Never
Filling out the history form	83(73.5%)	16(14.2%)	9(8%)	3(2.7)	21(1.8%)
Telemedicine/telephone interview	43(38.1%)	9(8%)	26(23%)	25(22.1%)	10(8.8%)
Face to face interview (interview in person)	85(75.2%)	23(20.4%)	4(3.5%)	1(0.9%)	0
Play with the child during assessment	35(31%)	39(34.5%)	32(28.3%)	5(4.4%)	2(1.8%)
Just being in the assessment room	31(27.4%)	26(23%)	35(31%)	19(16.8%)	2(1.8%)
No parental participation	1(0.9%)	2(1.8%)	18(15.9%)	39(34.5%)	53(46.9%)

***The effects of education/experience level of Iranian SLPs on choice of pre-assessment procedure*.** No significant relationship was found between level of education and experience for all three pre-assessment activities presented to SLPs. The majority of respondents reported that all three activities are used always and often by them.

#### Direct assessment

**Fig 1:**
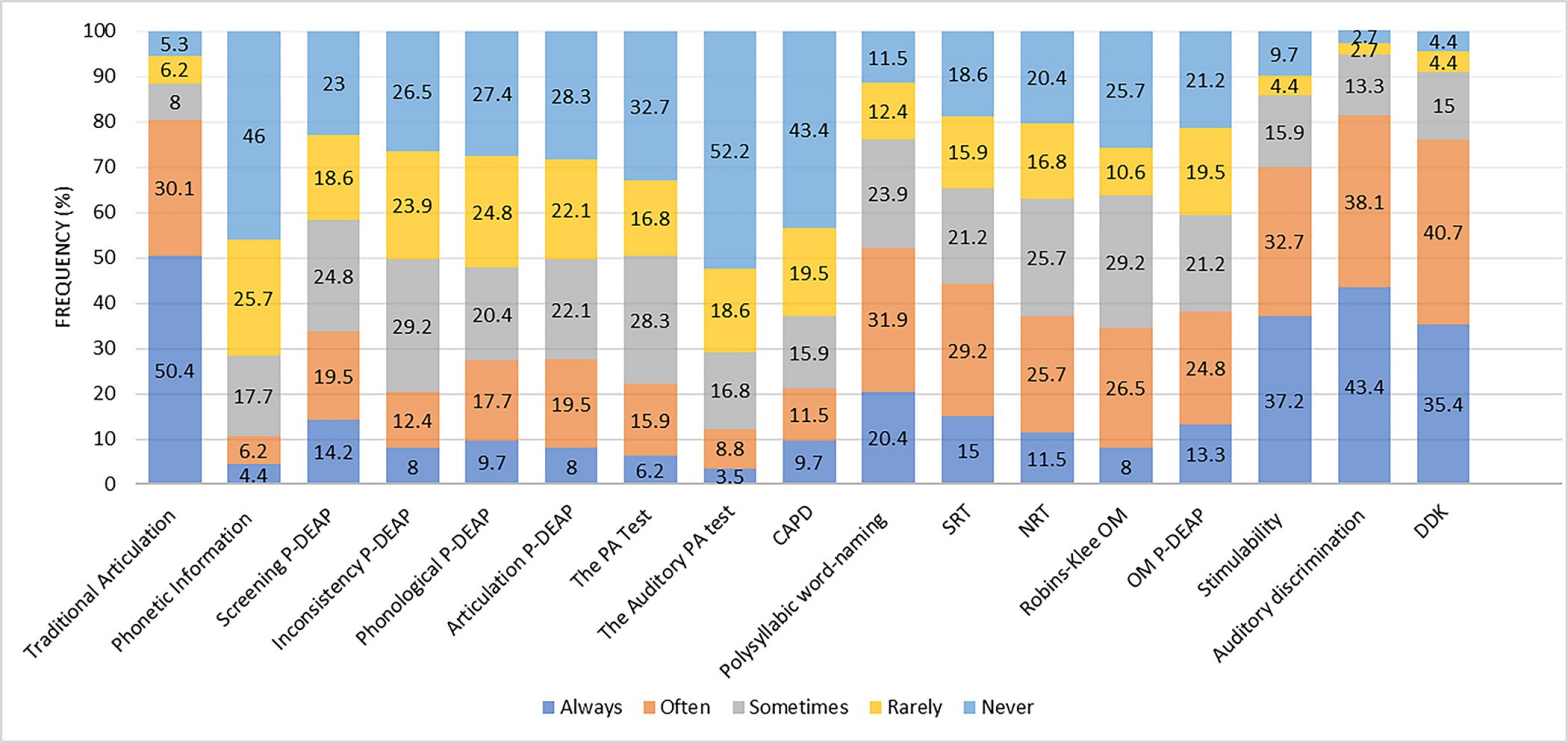
The frequency of direct assessment tools used by SLPs (N = 113). P-DEAP = Persian Diagnostic Evaluation of Articulation and Phonology, PA = Phonological Awareness, CAPD = Cleft Audit Protocol for Speech, SRT = Syllable Repetition Test, NRT = Nonword Repetition Test, OM = Oral-Motor, DDK = Diadochokinesis Test.

A list of 17 tests administered while interacting directly with children was presented to respondents. The TAT was favoured by more than half of the participants. Fig 1 summarises the frequency of use of these tools.

**Fig 2:**
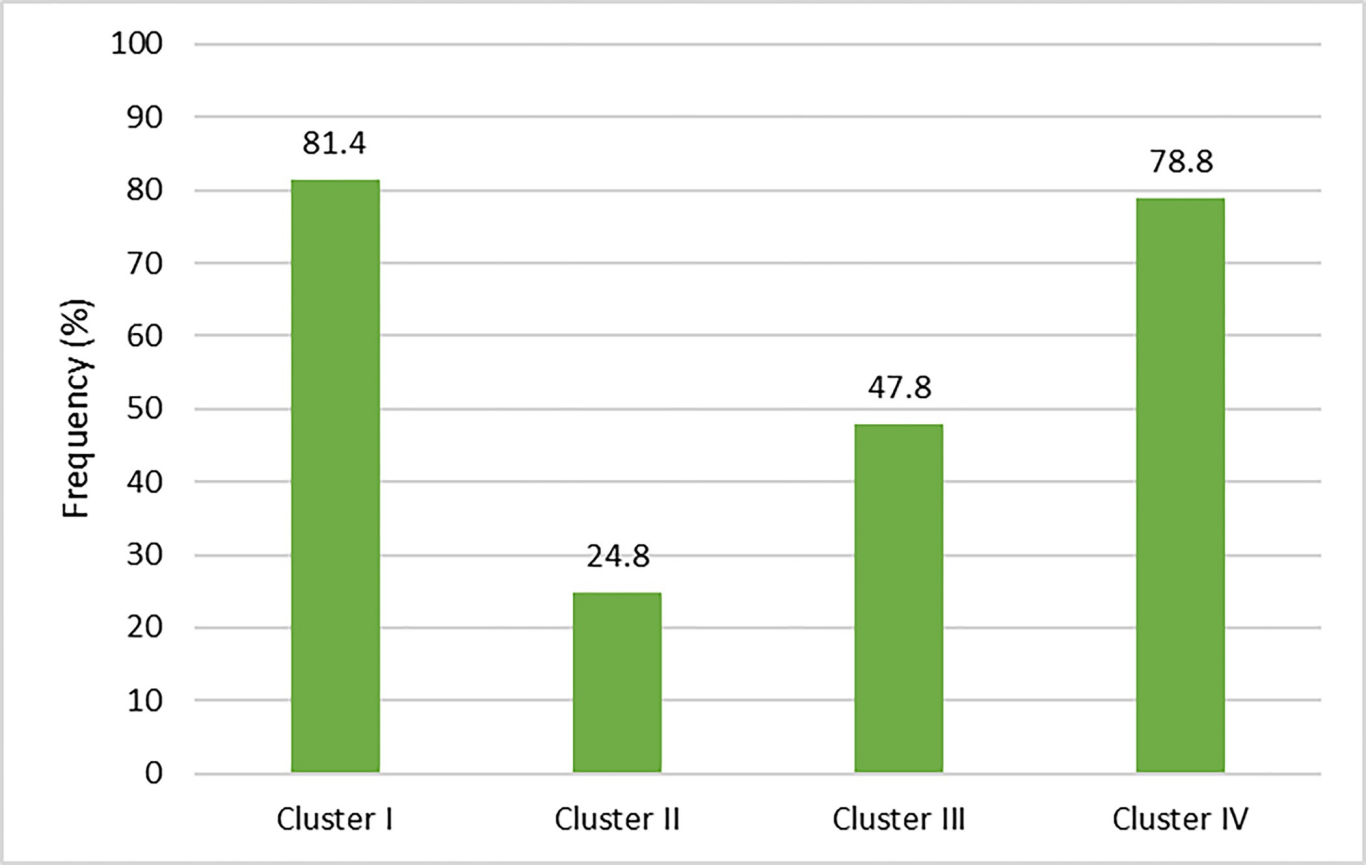
The frequency of chosen assessment tools clustered by type of task (n = 113). The cluster I includes: Traditional Articulation Test(TAT), Phonetic Information Test, Phonological Persian-DEAP Test, Phonetic Persian-DEAP Test, Polysyllabic Words Naming Assessment; the cluster II includes: The Phonological Awareness Test, The Auditory Test of Phonological Awareness Skills; the cluster III includes: Robins-Klee Oral Motor Assessment Protocol, Oral-Motor DEAP-Persian Test; the cluster IV includes: Cleft Audit Protocol for Speech, Syllable Repetition Test, Nonword Repetition Test, Inconsistency P-DEAP Test, Screening P-DEAP Test, Diadochokinetic Test, Auditory discrimination, Stimulability Test.

Assessments were grouped into four clusters based on their similarity. Cluster I included five picture naming tests, e.g., TAT, Phonetic Information Test, Phonological P-DEAP Test, Articulation P-DEAP Test, and Polysyllabic Words Test. Cluster II included two phonological awareness tools, e.g., the Phonological Awareness Test and Auditory Test of Phonological Awareness Skills. Cluster III consisted of two oral motor assessments, e.g., Robins-Klee Oral Motor Assessment Protocol and Oral-Motor P-DEAP Test. Cluster IV covers eight tests that assist in the diagnostic process including Nonword Repetition Test, Syllable Repetition Test, Cleft Audit Protocol for Speech, Inconsistency P-DEAP, the Screening P-DEAP, Auditory discrimination, Stimiulability test, and Diadokokinetic test. Fig 2 shows that picture naming tests (81.4%) were the most frequently used tools.

*The effects of education/experience level of Iranian SLPs on choice of direct assessment procedure*. A significant difference was found between two assessments (polysyllabic word-naming and Inconistency P-DEAP) and the level of experience. The experienced SLPs always and often (62.7%) chose polysyllabic word-naming (χ^2^ = 11.252, *p*<0.001), while the Inconistency P-DEAP was not used by the experienced respondents (χ^2^ = 7.927, *p* = 0.019); they rarely or never (89.9%) used Inconistency P-DEAP in assessing SSD. Although there was no significant relationship between other tests and level of experience, TAT was favoured by less-experienced SLPs and this inclination reduced as experience level increased. No significant difference between groups of respondents and selection of assessment clusters was found based on their level of experience (Table 4).

**Table 4:** The frequency of assessment clusters selected based on SLPs’ level of experience (*N* = 113).

	Cluster I (Picture word-naming tools) %		Cluster II (Phonological awareness tools) %		Cluster III (Oral-Motor tools) %		Cluster IV (Diagnostic support tools) %	
**Number of chosen tools**	**0 or 1**	**>2**	**0**	**1 or 2**	**0**	**1 or 2**	**0–3**	**>3**
Less-experienced	21.7	78.3	91.3	8.7	65.2	34.8	30.4	69.6
mid-experienced	25.8	74.2	71	29	41.9	58.1	9.7	90.3
Experienced	13.6	86.4	71.2	28.8	52.5	47.5	23.7	76.3

There was no significant association between hours of clinical activity per week and choice of direct assessments. However, those with few years of practice (1–3 years) used TAT significantly more than other groups (χ^2^ = 10.407, *p* = 0.015). A positive correlation exists between the administration of the DDK test and the number of years spent in practice. Individuals with a greater number of years of work experience are more likely to utilise the DDK test (r = 0.196, p = 0.038). No significant associations were found between the four assessment clusters and years of practice, but participants with less than five years of practice did not select any of the phonological awareness tests. Furthermore, cluster I tools were used least by respondents with more years of practice. SLPs with few years of practice reported more frequent use of assessments in the cluster III category. Comparison of mean education level and type of assessment showed significant use of TAT by those with bachelor’s degrees (93.9%) as a typical tool in assessing SSD (χ^2^ = 4.619, *p* = 0.032).

Respondents were asked to rate their level of confidence in applying tools for assessing SSD. Confident participants always and often (88.3%) implemented TAT. The majority of respondents with high confidence (81.1%) also administered two or more picture naming tests; 75.7% chose more than two diagnostic-assistant tools; 74.8% did not choose any of the cluster II tools and 51.4% reported never using cluster III assessments.

#### Post-assessment procedure

**Fig 3:**
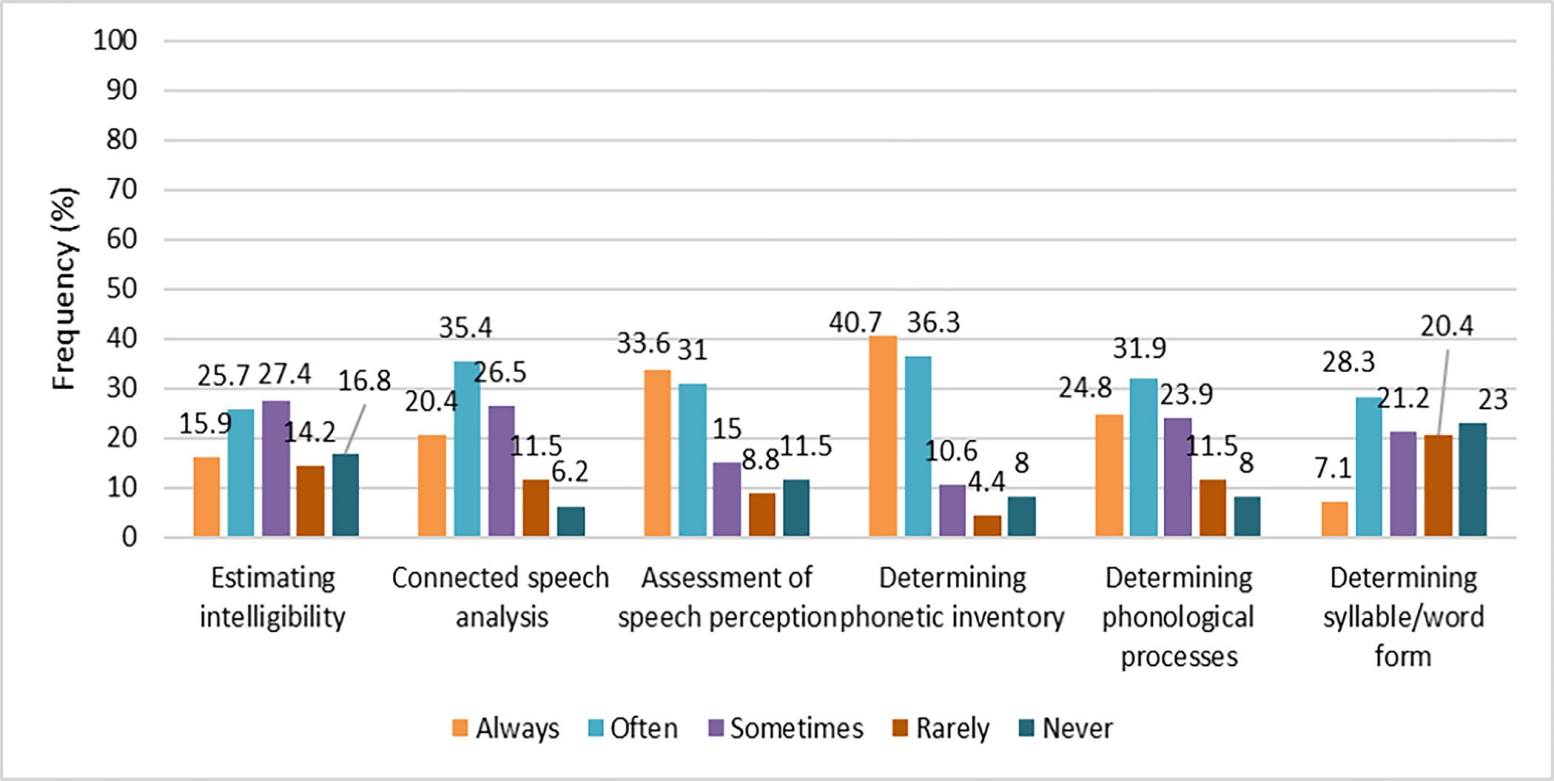
The frequency of post- assessment data analysis tasks used by 113 respondents (N = 113).

A list of six tasks was used when asking participants to specify the nature of their post-assessment procedure. Most participants determined the phonetic inventory (40.7%) and speech perception (33.6%) (Fig 3).

Three questions were asked about completing paperwork, the importance of writing notes and the challenges SLPs confront when writing clinical records. Although more than half of the participants stated that documentation is challenging, 92% reported writing clinical reports. Regarding the importance of documentation, 80.6% of SLPs attached high importance (very important + in most cases) to documentation. An open question was asked about the most important challenges the SLPs faced in their clinical documenting. The time-consuming nature of documentation was the most common challenge mentioned. Several clinicians identified a lack of a common framework of documentation among SLPs, nonspecific case sheets for different work settings and no unified electronic system for documentation, often leading to incomprehensible data. Printed documentation was not welcomed because of the high cost and extra space occupied in the clinic, making the maintenance and accessibility of the files difficult over the years. The last challenge was clinicians’ lack of knowledge and experience in recording quantitative measures of assessment results and therapeutic progress.

*Effects of education/experience level of Iranian SLPs on choice of post-assessment procedures*. There was no significant relation between the level of experience and post-assessment tasks and analyses. The utilisation of phonological process analysis and phonetic inventory determination is not contingent upon the level of experience of the participants. Indeed, nearly 80% of them employ these two analyses. The most frequently employed method among experienced SLPs was determining syllable/word form (62.7%).

The participants with a Bachelor’s degree demonstrated a greater propensity for utilising speech intelligibility estimation (72.7%) while those with a Master’s degree exhibited a proclivity for continuous speech analysis (91.7%), and syllable/word form determination was used more by those holding a PhD degree. Therefore, increasing use of post-assessment analysis was seen in respondents with higher levels of education. High confidence in clinical practice resulted in a high rate of speech supplementary tasks with 88.3% reporting phonetic inventory, 81.1% phonological process analysis and 56.8% syllable/word shape determination.

### Challenges of evaluating SSD

**Fig 4:**
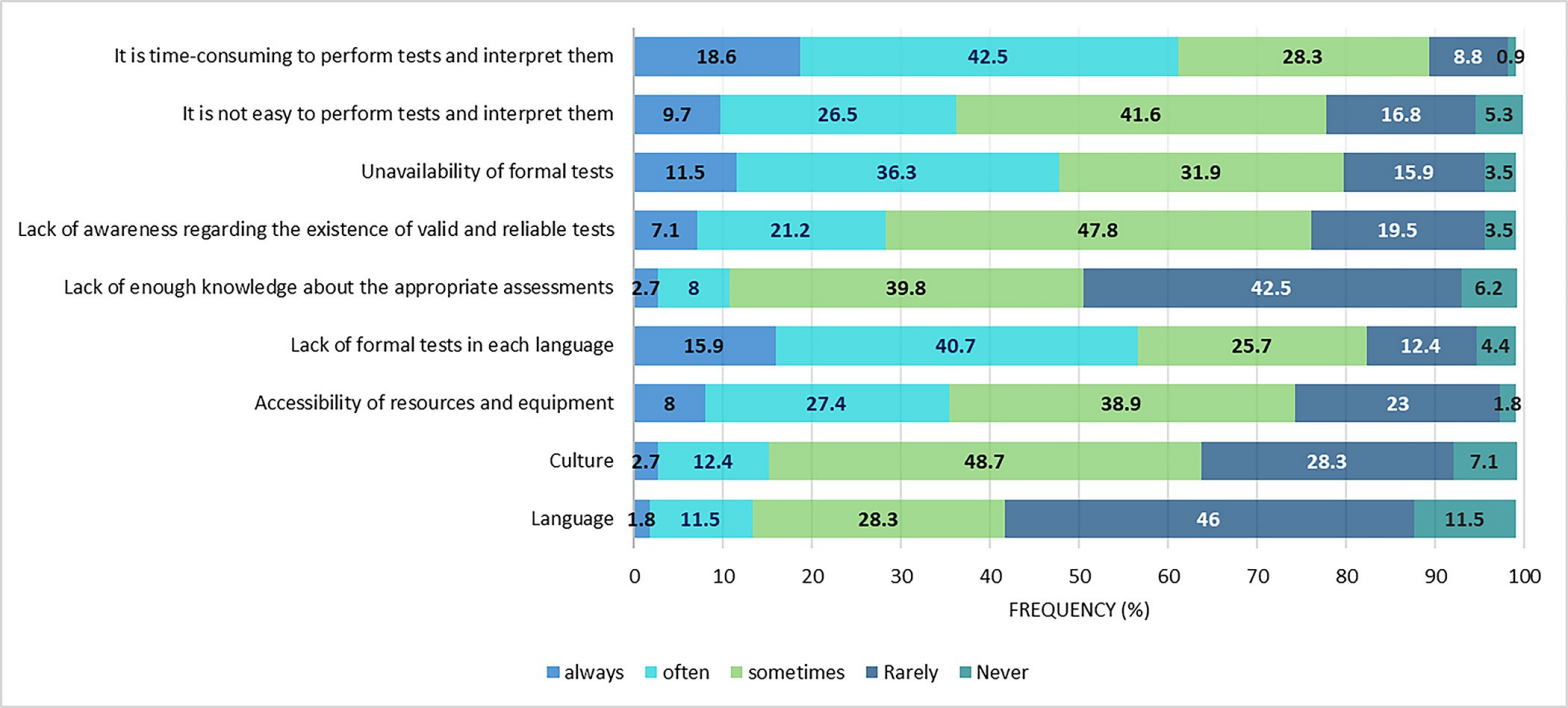
The frequency of the occurrence of the challenges during implementing assessments (N = 113).

The respondents were asked to rate the frequency of the challenges faced when implementing either formal or informal assessment of speech. Most respondents (18.6% always, 42.5% often) found the assessment procedures time-consuming (Fig 4).

### Keeping up to date with the evidence base

**Fig 5:**
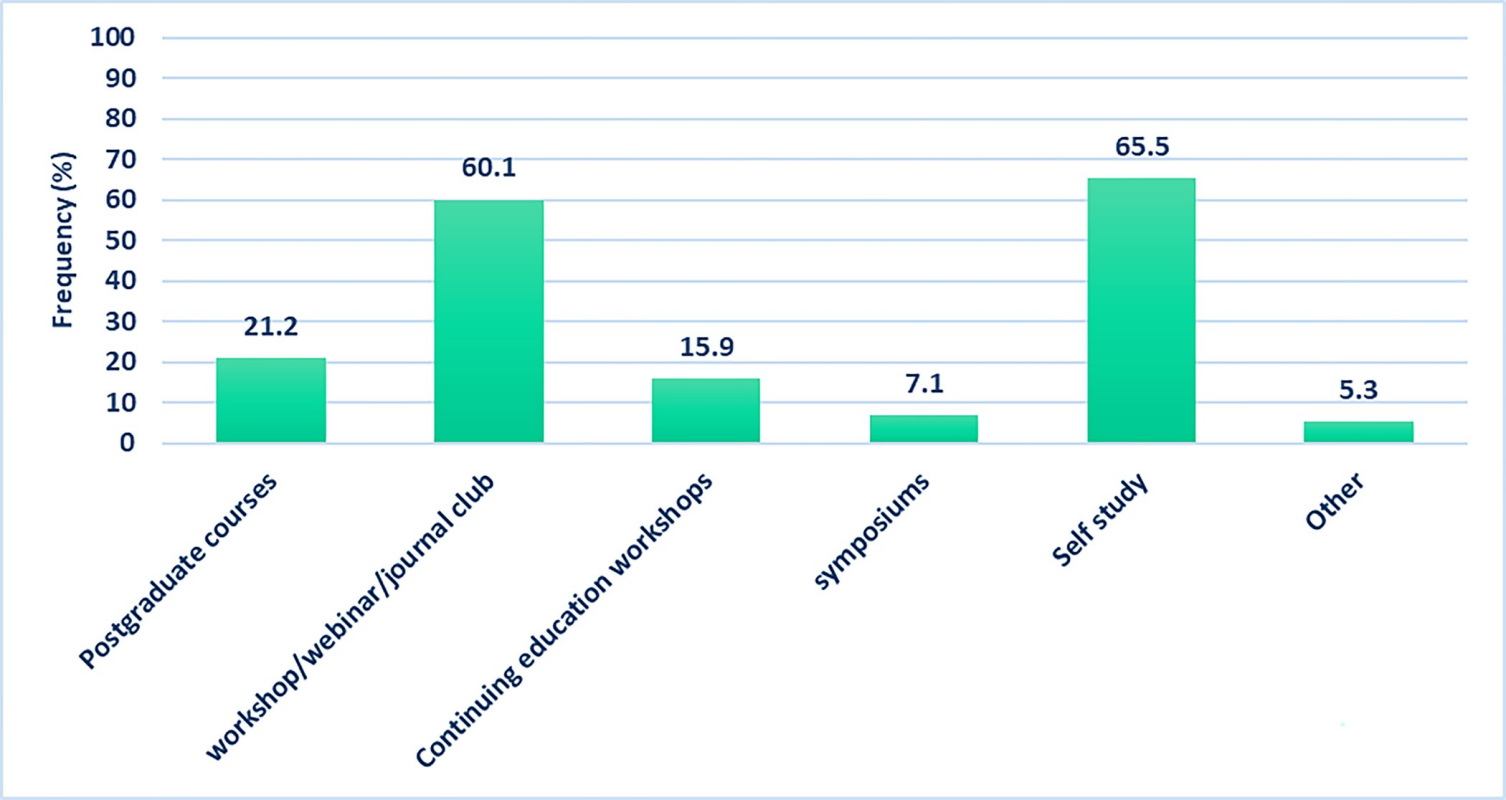
The frequency of study references (*N* = 113).

In response to the question “Which learning activity/ies resulted in most beneficial learning for you in the field of assessment of SSD?”, five sources were offered with multiple answers accepted. Self-study, workshop/webinars/journal clubs and postgraduate courses were the main ways of keeping up to date (Fig 5).

## Discussion

The study sought to describe the clinical experiences of Iranian SLPs regarding assessment of childen with SSD. The results provide useful information about clinicians’ routine practices in this field. We compare the findings of our research with findings from other non-English/English-speaking SLP surveys to understand similarities and differences in clinical practice.

### Service delivery

#### Language

All the respondents confirmed providing services in Persian, an expected result. A small proportion also offer services in Azeri Turkish (8%) and Kurdish (5%), reflecting the relative proportions of speakers of those languages [43]. Service provision in local languages seems appropriate but the availability and appropriate use of assessments in other languages–especially the three most recently released tests of Azeri [37], Kurdish [38] and Laki [36]–are not clear. This is consistent with reports of other studies of non-English language countries that lack appropriate tools, with informal measures often more favourable in these situations [14, 21]. Many English-speaking SLPs reported that they did not speak the first language of their clients and they used either standardised English assessments or informal assessments to assess children’s speech [12, 13].

#### Practice sites

The majority of our respondents work in private settings and governmental/university hospitals/clinics. This was consistent with reports of Australian SLPs [12] but contrasted with US SLPs [13] who see more children with SSD in schools. Most private practices in Iran specialise in areas of clinical practice and SSD assessment procedures are not conducted in settings like hospitals.

#### Caseload

SLPs in the US [13], Australia [12] and the UK [24] reported that children with SSD constitute more than half of their caseloads, whereas Iranian respondents reported a figure between 10–30%. Iranian parents may delay treatment for their children’s speech until they enter school, with the belief that speech difficulties will resolve on their own, based on cultural beliefs or lack of awareness about the benefits of early intervention.

### Assessing speech sound production

Assessment is the most important clinical activity of SLPs and the basis for determining a client’s needs [18, 44].

#### Pre-assessment procedures

Completing and reviewing case history forms was the most commonly performed activity reported by respondents. These were the most regular activities of US [13] and Australian SLPs [12], also undertaken through parent interviews. Dutch SLPs declared that the detailed description of problems by parents may help them to choose appropriate assessment procedures and establish a diagnosis [20].

Nearly half the respondents reported conducting hearing screening for their clients in the pre-assessment phase. In Skahan et al.’s study [13], hearing screening was reported as being frequently used, but this activity was reported as less common by Iranian, Australian [12] and Dutch SLPs [20]. These differences between the US and other countries in undertaking hearing screening may be related to the availability of audiologists and medical insurance rules to undertake hearing screening. For instance, according to Skahan et al., school nurses frequently collaborate with SLPs and often conduct hearing screenings in the US [13]. These findings do not necessarily suggest that SLPs do not value assessment of hearing status rather that they may find it difficult to arrange these assessments because only a referral from a paediatrician or otolaryngologist to an audiologist is covered by insurance, and all newborns undergo a hearing screening program at birth [45]. Thus, asking parents about their child’s hearing status based on neonatal screening results is the first option for Iranian SLPs instead of referring for a hearing screening test. However, preference for this approach carries the risk that children with conductive hearing loss due to otitis media may not be identified correctly and in a timely manner.

*Parental involvement in assessment procedure*. The most frequent activities of parents in assessment procedures were completing the history form and live interviewing, showing considerable agreement with previous reports [12, 13]. These two reported activities and other forms of involvement, e.g., accompanying the child into the assessment room, show a general tendency of SLPs to engage parents in assessment procedures [12–14].

#### Direct assessment

*Single*-word articulation assessments are the most popular tools administered in many settings [12–14, 20], comprising an essential part of the speech sound evaluation [18]. The TAT was the most frequently used single-word naming task by participants of the present study. The majority of Australian SLPs use single word naming tests to determine sounds in error but using such tests to determine standard scores is uncommon [12]. Previous surveys featured at least one formal naming instrument frequently used by participants [12–15, 20, 24], indicating that findings of the current study are inconsistent with other research. Unfortunately, the Iranian SLPs’ trend is utilising an informal and outdated tool for evaluating speech. In this test, three words are considered for each consonant, and there is only one possibility for the occurrence of the consonant in the initial, middle, and final position. As a result, there is no scoring system in place for this test. Rather, it is solely used to determine which error (substitution, deletion, distortion) has occurred. Furthermore, this test does not contain polysyllabic words, as well as various phonotactics and phonological process analysis, which would otherwise provide a richer data set. It can only be applied for providing phonetic inventory and may not reflect error patterns appropriately. It also cannot be used for differential diagnosis. The favouring of this test among clinicians with bachelor’s degrees revealed discrepancies and a knowledge gap in choosing pertinent assessment tools, along with lack of educational programs introducing valid and reliable assessments.

The inaccessibility and inappropriate distribution of assessment resources and lack of awareness of tools with appropriate psychometric properties in Persian may explain the lack of formal test use in Iran. However, workshops have been offered in different provinces to educate clinicians about reliable tests, e.g., P-DEAP. Another possible explanation for the frequent use of TAT may be time and work settings. Working in private settings and having large caseloads require tests that can be quickly administered.

Nearly three-quarters of the SLPs who were confident in their assessment choice, and those who had worked less than five years, do not use any of the phonological awareness tests. Phonological awareness is a valuable measure of the ability to manipulate the linguistic level of speech output and has a proven role in preventing later literacy problems [46], but it is not always included in assessment of children with SSD [13, 14, 20]. The low popularity of phonological awareness testing among Iranian clinicians is in line with other research. Newly graduated SLPs were expected to select evidence-based assessment tools given their recent formal training. Factors such as engaging in many different assessment procedures, time constraints and difficulty in reliable administration of phonological awareness tests before the age of four [14] may lead to the limited or incomplete use of phonological awareness testing in SSD evaluation procedures.

Similarly, oral-motor assessments were not used by more than 15%, and half of the confident respondents never use them. Our findings align with Swedish clinicians who reported using homemade materials and unspecified types of oral motor examinations [14], similar to SLPs in the Netherlands of whom only 10% reported using nonspeech oral motor movements as part of the diagnostic process [20]. In contrast, US clinicians use them frequently [13]. Preference for informal appraisals or observation of oral mechanism function–in addition to the unproven relationship between speech and nonspeech tasks [47, 48]–appear to be reasons for SLPs’ decisions to disregard oral-motor assessments to save time. However, oromotor assessments should help to differentially diagnose conditions such as submucous cleft palate, dysarthria and CAS. Thus, it seems the reasons and importance of using these assessment tools should be emphasised more for SLPs.

The Stimulability test was selected by the respondents as the third option. It is a test that is considered to provide valuable information for prognostic purposes, therapeutic decision-making and target selection [44], and also had high priority in previous surveys [12, 13].

#### Post-assessment procedure

Determining phonetic inventory was the most frequently used supplemental analyses reported by Iranian SLPs. Determining phonetic inventory was predictable in the current study given the high rate of selection of the TAT, which only provides phonetic information. The results were lower than expected (16%) for connected speech analysis, but the number of SLPs implementing this analysis increased with experience. Iranian SLPs–like Swedish SLPs [14]–elicit spontaneous speech and estimate intelligibility to a lesser degree than many clinicians in the US [13] and Australia [12]. Many SLPs believe in ecological validity and the importance of phonetic transcription of connected speech as an effective aspect of service delivery, although it is used to a limited extent in clinical practice. As a protocol for connected speech transcription of children with speech disorders is not yet available in Persian, limited use of this analysis was predicted. Iranian SLPs–like the Australian SLPs in Nelson’s [49] study, also appeared to have low confidence and time constraints regarding transcription and analysis of conversational sampling.

More than 90% of participants indicated their commitment to documentation, with more experienced respondents indicating greater awareness of the importance of paperwork. Skahan et al.’s [13] survey reported similar results for US SLPs. The main complaints of our respondents were the time-consuming nature of filling out case files and extensive paperwork in the absence of a unified electronic system of documentation. These complaints suggest that Iran’s health system should provide a standard framework for documenting the clinical information of patients in health centres.

### Challenges of evaluating SSD

Time-consuming tests, lack of formal Persian tools, few formal assessments, and lack of awareness about existing tools with psychometric properties in Persian were reported as the most frequent challenges of evaluating SSD in Iran. Lack of formal tools is a persistent complaint of non-English language SLPs who often end up using formal English test items informally with the children they see [14, 21, 22]. Time constraints [13], different requirements across work settings [20], and young children’s difficulties participating in long assessments [14] were affirmed by other researchers as critical issues impeding SLPs from completing comprehensive SSD assessments.

### Keeping up to date with the evidence base

Self-study and workshops/webinars were most frequently reported as ways for Iranian SLPs to remain up to date, consistent with results from other studies [12, 13]. These findings emphasise the importance of information resources like books, journals, and scientific databases, along with a need for developing appraisal guidelines and managing continuous educational programs through health discipline policies. There are many obstacles to remaining up to date in Iran, such as unavailability and inaccessibility of original assessment tools in different languages, restrictions in accessing digital resources, weak internet signals and political sanctions affecting research opportunities.

## Clinical implications

Iranian SLPs’ preference in SSD evaluation is implementation of informal, easily-administered tests–consistent with their colleagues around the world. However, based on two predominant systems of SSD classification [9, 50], we need assessments considering all aspects of the speech perception and production system for differential diagnosis, e.g., phonological error patterns, inconsistency and oral-motor assessments. Thus, there is a pressing need to design and present tools that can comprehensively evaluate specific characteristics of individual clients leading to appropriate therapeutic approaches.

## Limitations and future research

We faced several limitations during the current project. This study was designed during the COVID-19 pandemic with restrictions on public gatherings. As conferences and workshops were not held, a web-based questionnaire was prepared to distribute through available social media in Iran. Unfortunately, social media filtering restricted the availability of the questionnaire. Distributing the printed versions using the snowball sampling method was needed to gain sufficient data. Furthermore, published statistics of the number of working SLPs in healthcare services was not available to estimate the representativeness of SLPs’ participation.

This survey was designed to provide data regarding the experiences of SLPs in the SSD field. To keep a reasonable questionnaire length, we had to exclude some assessments and open-ended questions. Questions regarding the assessment time spent with each child in different settings and the assessment tools available in that setting, would also have been helpful. Further research with families from different ethnicities with multiple languages in Iran is needed to investigate the clinical experiences of SLPs dealing with bi/multilingual children even though this issue is still in its infancy worldwide.

## Conclusions

The study elucidates viewpoints and clinical experiences of Iranian SLPs working with children with speech disorders. The popularity of an informal phonetic test revealed that a significant proportion of Iranian SLPs do not adhere to global best practice recommendations. However, many similarities in the reported assessment procedures were found with international SLPs. Limited use of formal assessments in this study may reflect the clinicians’ lack of knowledge about using newly-adapted speech assessments in the local context or choosing assessments based on their personal experience. The findings indicate the need to develop more tools more able to deal with different aspects of the phonological system. It seems that national health policy should be directed to support investments in producing more easy and applicable tools e.g., digital resources to be distributed equitably throughout the country, and providing more convenient accessibility to existing tools. More importantly, there is a need to develop clinical guidelines for evaluating SSD in the Persian language.

Our findings revealed a need to keep up to date based on evidence, along with advancement of dynamic continuing education programs. The problems mentioned by SLPs in using assessment tools may be due to limited financial resources/investments or less technical equipment, along with the inequitable distribution of resources, failure in widening knowledge and expansion of educational opportunities, and dissemination of scientific knowledge worldwide.

The resemblance in service provision in a developing country such as Iran with developed countries (e.g., the United States, Australia, Sweden, and the Netherlands) may make Iranian SLPs more confident and motivated towards their clinical activities. The variability in current data with respect to service provision should reinforce the SLP community’s need to address mandatory criteria and effective practice-based research in evaluation of SSD, reducing risk of literacy problems, and ensuring appropriate services for all children with SSD.

## Supporting information

S1 Appendix The questionnaire of clinical experiences of Iranian SLPs in managing SSD. (DOCX)

## List of abbreviations

SSD: Speech sound disorder
SLP: Speech-Language Pathologists
P-DEAP: Persian version of Diagnostic evaluation of articulation and phonology
TAT: Traditional articulation test
DDK: Diadochokinetic
