# Supplementary material for: Assessment of speech sound disorders: Clinical experiences of Speech-language pathologists in Iran
Source: PLoS One. 2024 Dec 16;19(12):e0310885. doi: 10.1371/journal.pone.0310885 (PMC11649114; doi:10.1371/journal.pone.0310885)
Supplement: S1 Appendix — (DOCX) [file pone.0310885.s001.docx]

**Examining the experience of Iranian speech and language pathologists: Questionnaire about assessment and treatment of Speech Sound Disorder**

Dear colleague,

As you know, Speech Sound Disorder (SSD) is one of the most common speech and language disorders in children and there is always a large number of clients with SSD in speech therapy clinics. Since there is no information on how to diagnose, assess and treat children with SSD in our context, a research project entitled **"Examining the experience of Iranian speech and language pathologists: Questionnaire about assessment and treatment of Speech Sound Disorders**" is taking place in the University of Social Welfare and Rehabilitation Sciences. The questionnaire aims to obtain evidence about assessment and treatment processes for SSD in Iran by taking advantage of your valuable opinions for appropriate planning to improve the educational level and increase the clinical skills of therapists in this field. Your information will be kept completely confidential by the research team. The questions either require short answers or selection from multiple options, and we hope that you will answer these completely and honestly. Consent will be assigned by completing this questionnaire.

We kindly appreciate your participation in conducting this research

T. Zarifian, M. Imani, M Fotuhi

Department of Speech therapy

University of Social Welfare and Rehabilitation Sciences

1. ***Demographic Information***
2. Gender: man woman
3. Enter the year in which you received each document

| Educational certificate | Graduation year | Specify your field of study |
| --- | --- | --- |
| Bachelor's degree |  |  |
| Master’s degree |  |  |
| PhD |  |  |

1. **Write the number of years you have worked as a clinical Speech Therapist**…......... years
2. **On average, how many hours a week do you engaged in Speech therapy?** ........ hours
3. **Which of the following is your main field of expertise?** (It is possible to select more than one answers)

- Phonological disorders (phonological delay/ consistent and inconsistent Phonological disorders)
- Articulation (phonetic) disorders
- Childhood Dysarthria
- Adult Dysarthria
- Childhood apraxia of speech
- Developmental Language Disorders
- Speech fluency disorders
- Reading and writing disorders
- Hearing impairment
- Cleft palate
- Other, please explain if you work in other areas such as autism spectrum disorders, or adults.

1. **Which of the following options best describes the age group of people you work with (you can select more than one option)?**

Children under 3 , Preschoolers , Primary-schoolers , Adults , Other (……...)

1. ***Service Delivery***
2. **In which province/city do you work? ......................................**
3. **What languages do you provide services in?** ..................................
4. **Have you mastered the language of the region where you carry out speech therapy services?.....................................**
5. **Do you work with bilingual (multilingual) children? If the answer is yes, please mark the languages.** (It is possible to choose more than one answer).

| - Persian | - Turkish | - Gilaky | - Kurdish |
| --- | --- | --- | --- |
| - Mazani | - Arabic | - Lori | - English |
| - Other (please explain):   **11. What action do you take if a child with SSD who speaks another language comes to you and you are not familiar with his mother tongue? (**It is possible to choose more than one answer).   - I get help from speech therapist colleagues who have mastered that language. - I get help from the child's family members. - I have not had such clients so far and have not done such an assessment. - I do not accept such clients and refer them to a speech therapist who has mastered the language. - Other (please write down whatever you do) ........................................................... | | | |

1. **In which setting(s) do you work? (**It is possible to choose more than two answers.)

| - private office - Private hospital | - Governmental school - private school |
| --- | --- |
| - Governmental clinic/hospital affiliated with governmental organizations - state clinics affiliated with universities | - (comprehensive) daily rehabilitation centers - Non-governmental organizations (NGO) such as the Autism Association or the Down Syndrome Association |
| - Other (please explain) | |

1. **What percentage of your clinical records are related to children AND adults with speech sound disorders (articulation, phonological including delay, consistent and inconsistent, dysarthria, apraxia)**

- Less than 10%
- 10% to 30%
- 40% to 70%
- Up to 7

***C) Assessment***

***C1) pre-assessment procedure***

1. **Indicate the extent to which you use the activities listed in the table below.** Please select an option for each line of the table.

|  | **always** | **Often** | **sometimes** | **rarely** | **never** |
| --- | --- | --- | --- | --- | --- |
| Interview with parents |  |  |  |  |  |
| Child history |  |  |  |  |  |
| Auditory screening |  |  |  |  |  |
| Other (write it down if used): |  |  |  |  |  |

***parents participation***

1. **When assessing children, in which of the following areas do parents participate**

**in the assessment?** Please select an option for each line of the table.

|  | **always** | **often** | **sometimes** | **rarely** | **never** |
| --- | --- | --- | --- | --- | --- |
| Completing the history form |  |  |  |  |  |
| Telemedicine/telephone interview |  |  |  |  |  |
| Live interview (interview in person) |  |  |  |  |  |
| Play with the child during assessment |  |  |  |  |  |
| Just being in the assessment room |  |  |  |  |  |
| There is no parents’ participation |  |  |  |  |  |

***C2) Direct assessment***

1. **Determine how much you use each of the following tests:** Please select an option for each line of the table.

|  | **always** | **often** | **sometimes** | **rarely** | **never** |
| --- | --- | --- | --- | --- | --- |
| Traditional phonetic test (TAT) |  |  |  |  |  |
| Phonetic information test, Ghasisin et al. |  |  |  |  |  |
| Screening P- DEAP test |  |  |  |  |  |
| Inconsistency P-DEAP test |  |  |  |  |  |
| Phonological P-DEAP test |  |  |  |  |  |
| Phonetic P-DEAP test |  |  |  |  |  |
| Oral-Motor P-DEAP test |  |  |  |  |  |
| Robbins-klee Oral Motor Assessment Protocol |  |  |  |  |  |
| Assessment of phonological awareness skills (Soleimani, et al.) |  |  |  |  |  |
| Assessment of phonological awareness skills (Kashani) |  |  |  |  |  |
| Cleft Audit Protocol for Speech (CAPS) |  |  |  |  |  |
| Diadochokinetic test (DDK) |  |  |  |  |  |
| Polysyllabic word naming assessment |  |  |  |  |  |
| Syllable Repetition Test (SRT) |  |  |  |  |  |
| Non-word Repetition Test (NRT) |  |  |  |  |  |
| Auditory discrimination |  |  |  |  |  |
| Stimulability test |  |  |  |  |  |

***C3) Post-assessment assessment***

1. **Determine how much you use each of the following postassessment acrivities:** Please select an option for each line of the table.

|  | **always** | **often** | **sometimes** | **rarely** | **never** |
| --- | --- | --- | --- | --- | --- |
| Evaluation of speech intelligibility (determining the percentage of speech intelligibility) |  |  |  |  |  |
| Connected speech analysis |  |  |  |  |  |
| Assessment of speech perception |  |  |  |  |  |
| Determining phonetic inventory |  |  |  |  |  |
| Determining phonological processes |  |  |  |  |  |
| Determining syllable/word form |  |  |  |  |  |
| Other |  |  |  |  |  |

1. **Do you have clinical records for your clients?** Yes No
2. **How important do you consider writing clinical records?**

- most important
- often important
- somewhat important
- not important too much
- never important

1. **How challenging is writing clinical records for you?**

- Very much
- Much
- Neither too low nor too much
- Low
- Very low

Please write your most important challenge in document writing ......................................................

1. **What are your biggest challenges in assessment (formal/informal) of children**

**with speech impairments?** Please select an option for each line of the table.

|  | **always** | **often** | **sometimes** | **Rarely** | **Never** |
| --- | --- | --- | --- | --- | --- |
| Language |  |  |  |  |  |
| Culture |  |  |  |  |  |
| Availability of resources and equipment |  |  |  |  |  |
| Lack of formal tests in each language |  |  |  |  |  |
| Lack of enough knowledge about appropriate assessments in this area |  |  |  |  |  |
| Lack of knowledge of existing assessments that are valid and reliable for use in Persian or common languages in Iran |  |  |  |  |  |
| Unavailability of formal tests |  |  |  |  |  |
| It is not easy to perform tests and interpret them |  |  |  |  |  |
| It is time-consuming to perform tests and interpret them |  |  |  |  |  |
| Other (please explain): |  |  |  |  |  |

1. **To what extent are you confident about the assessments that you have chosen to examine the speech sound disorder of your clients?**

- Very much
- Much
- Neither too low nor too much
- Low
- Very low

1. **Which learning activity/ies resulted in most beneficial learning for you in the field of *Assessment* of SSD?** It is possible to select more than one answer

- Undergraduate courses
- Postgraduate courses
- Participation in workshop/webinar/journal club
- Continuing education workshops of universities/government organizations
- attending in the symposiums
- Independent study
- Other

***E) Intervention/Treatment***

1. **Based on which of the following items do you select your target treatment? Please use the scale below to indicate how often you use each approach in treatment**

|  | always | often | sometimes | rarely | never |
| --- | --- | --- | --- | --- | --- |
| Formal assessments results |  |  |  |  |  |
| Informal assessment results |  |  |  |  |  |
| Family opinions and preferences |  |  |  |  |  |
| Targets that facilitate the child's early success |  |  |  |  |  |
| Age order of the sounds’ emergence in typically developing children |  |  |  |  |  |
| choosing a phonological process or error patterns that leads to significant intelligibility in speech |  |  |  |  |  |
| Sounds stimulability |  |  |  |  |  |
| high frequency speech sounds in the language |  |  |  |  |  |
| Less-complex Speech sounds in terms of production |  |  |  |  |  |
| A target that is meaningful and functional for the child |  |  |  |  |  |

1. **Below is a list of common treatment methods for speech sound disorders. Please use the scale below to indicate how often you use each approach in treatment**

| **Treatment approach** | **always** | **often** | **sometimes** | **rarely** | **Never** |
| --- | --- | --- | --- | --- | --- |
| Minimal pair treatment |  |  |  |  |  |
| Maximal opposition treatment |  |  |  |  |  |
| Multiple oppositions treatment |  |  |  |  |  |
| Cycle approach |  |  |  |  |  |
| Traditional articulation treatment (Van Riper) |  |  |  |  |  |
| Non-speech oromotor practices |  |  |  |  |  |
| Core vocabulary treatment |  |  |  |  |  |
| Myofunctional therapy/tongue thrust therapy |  |  |  |  |  |
| Metaphon therapy |  |  |  |  |  |
| Phonological awareness |  |  |  |  |  |
| Psycholinguistic approaches |  |  |  |  |  |
| Parent-directed approaches |  |  |  |  |  |
| Combination of several treatment methods (e.g., cycle + Phonological awareness) |  |  |  |  |  |
| Motor approaches based on sensory-motor (PROMPT, DTTC) |  |  |  |  |  |
| Other |  |  |  |  |  |

1. **Please indicate if you have ever used these treatment methods and in which way (individual, group, combined (individual-group))?** Please select an option for **each** line of the table.

| **Treatment approaches** | **individual** | **group** | **combined (individual-group)** |
| --- | --- | --- | --- |
| Minimal pair treatment |  |  |  |
| Maximal opposition treatment |  |  |  |
| Multiple oppositions treatment |  |  |  |
| Cycle approach |  |  |  |
| Traditional articulation treatment (Van Riper) |  |  |  |
| Non-speech oromotor practices |  |  |  |
| Core vocabulary treatment |  |  |  |
| Myofunctional therapy/tongue thrust therapy |  |  |  |
| Metaphon therapy |  |  |  |
| Phonological awareness |  |  |  |
| Psycholinguistic approaches |  |  |  |
| Parent-directed approaches |  |  |  |
| Combination of several treatment methods (e.g., cycle + Phonological awareness) |  |  |  |
| Motor approaches based on sensory-motor (PROMPT, DTTC) |  |  |  |
| Other |  |  |  |

1. **On average, how long are your treatment sessions in treating children with SSD? ……. Minutes**
2. **How many times a week do you usually hold your therapy sessions?**

- Once a week
- Twice a week
- Three times a week
- more than three times a week
- others

1. **How effective have you found intensive treatment sessions (more than once a week) based on your experience?**

- Very effective
- Effective
- I don’t know
- Ineffective
- Very ineffective

1. **Do you get help from the child’s parents or caregivers/nurse to practice at home?**

- Always
- Often
- Sometimes
- Rarely
- Never

1. **Please score the effectiveness of each of the following treatment methods from 1 (lowest) and 5 (highest).** Please select an option for **each** line of the table.

| **Treatment methods** | **1** | **2** | **3** | **4** | **5** | **Haven’t used the approch yet** |
| --- | --- | --- | --- | --- | --- | --- |
| Minimal pair treatment |  |  |  |  |  |  |
| Maximal opposition treatment |  |  |  |  |  |  |
| Multiple oppositions treatment |  |  |  |  |  |  |
| Cycle approach |  |  |  |  |  |  |
| Traditional articulation treatment (Van Riper) |  |  |  |  |  |  |
| Non-speech oromotor practices |  |  |  |  |  |  |
| Core vocabulary treatment |  |  |  |  |  |  |
| Myofunctional therapy/tongue thrust therapy |  |  |  |  |  |  |
| Metaphon therapy |  |  |  |  |  |  |
| Phonological awareness |  |  |  |  |  |  |
| Psycholinguistic approaches |  |  |  |  |  |  |
| Parent-directed approaches |  |  |  |  |  |  |
| Combination of several treatment methods (e.g., cycle + Phonological awareness) |  |  |  |  |  |  |
| Motor approaches based on sensory-motor (PROMPT, DTTC) |  |  |  |  |  |  |
| Other |  |  |  |  |  |  |

1. **Specify the amount of use of each type of feedback in the following items.** Please select an option for **each** line of the table.

|  | **always** | **often** | **sometimes** | **rarely** | **Never** |
| --- | --- | --- | --- | --- | --- |
| Linguistic feedback (Using minimal pairs in meaningful questions. For example, I don't think the boy drinks key, we drink tea, we don't drink key) |  |  |  |  |  |
| Visual or auditory-verbal feedback about phonetic placement |  |  |  |  |  |
| Visual and tactile imagination |  |  |  |  |  |
| Request for self-monitoring or self-correction |  |  |  |  |  |
| Repeating the child's error to get his/her self-awareness |  |  |  |  |  |
| Token reinforcement |  |  |  |  |  |
| Praising the child for good effort and participation in activities |  |  |  |  |  |
| Feedback to performance |  |  |  |  |  |
| Feedback to the results |  |  |  |  |  |
| Immediate feedback |  |  |  |  |  |
| Delayed feedback |  |  |  |  |  |
| Random feedback |  |  |  |  |  |

1. **How effective do you think providing appropriate feedback in therapy sessions is for the child's progress based on your experience?( You can select more than one answer).**

- I have used all kinds of feedback in treatment and I find it **very** useful.
- I find feedback on performance, especially on the place and manner of articulation, **very** useful.
- Feedback to the result of speech sound productions, random and delayed feedback are **very** beneficial.
- I have not been trained enough in university on how to give feedback.
- I have used various types of feedback in therapy, but children do not respond to feedback and it is not useful for them.

1. **How confident are you about your ability and adequacy in implementing the selected treatment approach?**

- Very much
- Much
- Neither too low nor too much
- Low
- Very low

1. **Declare your agreement or disagreement with the following sentence:**

“There is enough evidence about the effectiveness of therapeutic interventions in children with speech sound disorders in Persian language”.

- Totally agree
- Agree
- I have no opinion
- Disagree
- Totally disagree

**36. Which item(s) resulted in proper training and learning for you in the field of treating SSD?** It is possible to select more than one answer.

- Undergraduate courses
- Postgraduate courses
- Participation in workshop/webinar/journal club
- Continuing education workshops of universities/government organizations
- attending in the symposiums
- Independent study
- What support/further training you would like when working with children with SSDs?
